# Metformin and Insulin Resistance: A Review of the Underlying Mechanisms behind Changes in GLUT4-Mediated Glucose Transport

**DOI:** 10.3390/ijms23031264

**Published:** 2022-01-23

**Authors:** Rok Herman, Nika Aleksandra Kravos, Mojca Jensterle, Andrej Janež, Vita Dolžan

**Affiliations:** 1 Department of Endocrinology, Diabetes and Metabolic Diseases, University Medical Center Ljubljana, 1000 Ljubljana, Slovenia; rokherman2@gmail.com (R.H.); nikakravos@gmail.com (N.A.K.); mojcajensterle@yahoo.com (M.J.); andrej.janez@kclj.si (A.J.); 2 Department of Internal Medicine, Faculty of Medicine, University of Ljubljana, 1000 Ljubljana, Slovenia; 3 Pharmacogenetics Laboratory, Institute of Biochemistry and Molecular Genetics, Faculty of Medicine, University of Ljubljana, 1000 Ljubljana, Slovenia

**Keywords:** metformin, insulin resistance, glucose transport, glucose transporter 4

## Abstract

Metformin is the most commonly used treatment to increase insulin sensitivity in insulin-resistant (IR) conditions such as diabetes, prediabetes, polycystic ovary syndrome, and obesity. There is a well-documented correlation between glucose transporter 4 (GLUT4) expression and the level of IR. Therefore, the observed increase in peripheral glucose utilization after metformin treatment most likely comes from the induction of GLUT4 expression and its increased translocation to the plasma membrane. However, the mechanisms behind this effect and the critical metformin targets are still largely undefined. The present review explores the evidence for the crucial role of changes in the expression and activation of insulin signaling pathway mediators, AMPK, several GLUT4 translocation mediators, and the effect of posttranscriptional modifications based on previously published preclinical and clinical models of metformin’s mode of action in animal and human studies. Our aim is to provide a comprehensive review of the studies in this field in order to shed some light on the complex interactions between metformin action, GLUT4 expression, GLUT4 translocation, and the observed increase in peripheral insulin sensitivity.

## 1. The Role of GLUT4 Expression in the States of Insulin Resistance

Several chronic conditions accompanied by insulin resistance (IR) as their main unifying characteristic have been on a steady incline over the last decades [1,2]. Our understanding of insulin-stimulated glucose transport has advanced enormously over time, from the first report in 1939 that insulin stimulates glucose uptake into rodent muscle, to the comprehension of the importance of glucose transporter translocation after insulin stimulation, to later findings that many different transporters regulate glucose transport into cells [1,3]. In 1988, James et al. provided the first evidence of the antigenically unique insulin-regulatable glucose transporter protein, which is now widely known as glucose transporter 4 (GLUT4) and is encoded in humans by the solute carrier *SLC2A4* gene [4]. In addition to providing considerable insight into vesicle transport and the insulin-signaling pathway, the discovery of GLUT4 has advanced our comprehension of tissue and organ metabolism [1]. With the later findings that GLUT4-mediated transmembrane glucose transport is the rate-limiting step in peripheral glucose utilization and that the level of glucose transporter directly influences glucose disposal in target tissues, the understanding of insulin-stimulated glucose uptake and the role of its deterioration in the states of IR advanced rapidly [5,6,7,8,9].

In fact, many animal and human studies have indicated that impaired GLUT4-mediated glucose uptake is the primary underlying mechanism of IR. For example, in obese hypertensive rats, representing an animal model of metabolic syndrome, Leguisamo et al. found a reduction in GLUT4 expression accompanied by whole-body IR [10]. Similarly, about 50% lower GLUT4 levels were noted in skeletal muscle of hypertriglyceridemic IR rats compared to normal Wistar rats [11]. In a study on diabetic mouse adipocytes, GLUT4 levels were reduced by 34%, and a 46% reduction in insulin-stimulated glucose transport was observed [12].

Studies in transgenic mouse models facilitated an even better understanding of the role of GLUT4 expression in IR. A 70% decrease in GLUT4 protein expression and a 72% reduction in insulin-stimulated glucose transport resulted from the adipose-specific genetic knockout of GLUT4 [13]. Moreover, the model in this study exhibited IR and glucose intolerance, with significant additional defects in insulin-stimulated glucose uptake in skeletal muscle and insulin-mediated suppression of glucose production in the liver [13]. The same systemic effect of tissue-specific GLUT4 disruption was noted in another experiment by the same research group, in which muscle-specific knockout of GLUT4 expression led to IR in adipose tissue and liver [5]. The research group that conducted both experiments concluded that regardless of which tissue harbors the primary genetic defect of the GLUT4 expression, the other insulin target tissues are ultimately affected [14]. Additional studies on GLUT4 heterozygous knockout mice also showed the development of IR in adipose and muscle tissue and significant enhancement in the likelihood of diabetes development [15,16,17].

On the other hand, the opposite effect was demonstrated in studies on transgenic mice overexpressing GLUT4. In models overexpressing GLUT4 in adipose tissue, a 2-fold in vivo increase in insulin-stimulated glucose transport was noted [18]. Furthermore, Carvalho et al. discovered that overexpression of GLUT4 in adipose tissue of mice with muscle GLUT4 knockout normalized fasting hyperglycemia and glucose intolerance without restoring glucose transport in muscle [19]. Two additional studies came to the same conclusions, but overexpression of GLUT4 improved glucose tolerance and insulin sensitivity in normal as well as genetically diabetic mice [20,21]. Moreover, transgenic mice engineered to express the human GLUT4 gene and promoter remained highly insulin sensitive on a high-fat diet compared to their nontransgenic counterparts [22].

Human studies have complemented the results obtained in animal models and further emphasized the role of GLUT4 in IR [1]. In 1991, Garvey et al. observed significantly lower GLUT4 mRNA levels in adipose tissue of obese subjects compared to lean controls, and they were even further reduced in the non-insulin-dependent type 2 diabetes mellitus (T2DM) group [23]. Two years later, Rosenbaum et al. published a paper in which they reported that the maximal insulin-stimulated increment in adipocyte glucose transport was independently decreased by obesity and polycystic ovary syndrome (PCOS) compared to controls [24]. In both conditions, GLUT4 content in adipocyte membranes was reduced, and there was a high correlation between the GLUT4 content and the maximum rate of insulin-stimulated glucose uptake in adipocytes [24]. Garvey et al. also studied nondiabetic and diabetic subgroups and demonstrated that human IR involves a defect in GLUT4 trafficking and targeting, leading to the inability of insulin to recruit GLUT4 to the plasma membrane [25]. Furthermore, another study demonstrated the relationship between the severity of IR and GLUT4 expression in skeletal muscle of T2DM patients [26].

Lifelong physical activity was also shown to prevent aging-associated IR in human skeletal muscle myotubes by increasing GLUT4 mRNA and protein expression as well as its translocation ability [27]. Another example of the role of GLUT4 in IR was a study in young, healthy male subjects who completed seven days of bed rest and showed significant IR in muscle biopsies that mainly occurred through reduced GLUT4 content [28]. More recently, Ezeh et al. noted that patients with PCOS had higher scores on the homeostatic model assessment for IR (HOMA-IR) and a trend toward lower GLUT4 mRNA expression in adipose tissue than the healthy control participants [29]. In another paper, the same research group also compared GLUT1 and GLUT4 mRNA levels in adipocytes of PCOS women and matched controls. Their results suggest that IR secondary to lower insulin-mediated glucose uptake and enhanced insulin secretion in PCOS is partly attributable to a reduction in adipocyte GLUT4 expression that is not accompanied by a compensatory increase in GLUT1 expression [30].

## 2. The Effect of Metformin Treatment on Insulin Resistance and GLUT4 Expression

Metformin is a widely prescribed insulin-sensitizing agent in current clinical use. Despite being most commonly associated with its ability to decrease plasma glucose levels, the knowledge about its novel properties and effects continues to evolve [31]. Its action modulates multiple biological pathways. Regarding glucose homeostasis, it mainly works by improving insulin-mediated suppression of hepatic glucose production and enhancing insulin-stimulated glucose disposal in peripheral tissues; however, the precise molecular mechanisms of this action remain elusive [32]. By deploying different measurements for insulin sensitivity in humans, metformin has been shown to improve whole-body sensitivity to insulin in numerous studies in different IR conditions and study groups [33,34,35,36,37,38,39,40,41,42,43].

Experimental studies have shown that metformin-mediated improvements in insulin sensitivity might be associated with several mechanisms, including increased insulin receptor tyrosine kinase activity, enhanced glycogen synthesis, and at the most downstream end, an increase in the recruitment and activity of GLUT4 [32]. These effects may be achieved through direct and indirect mechanisms [32,44]. Its effect on several steps in the insulin signaling cascade involved in the generation and propagation of the insulin signal could be one mechanism of overcoming molecular defects of insulin action in IR [32]. Additionally, through inhibition of mitochondrial complex I and other potential pathways, metformin may activate adenosine 5′-monophosphate-activated protein kinase (AMPK), a so-called cellular energy sensor [44,45]. It also has the ability to stimulate glucagon-like-peptide-1 release, thereby enhancing insulin secretion and lowering plasma glucose levels [46]. Moreover, recent studies suggested that gut microbiota may be one additional target site [47].

Many studies have noted that metformin affects GLUT4 expression, translocation, and function but have not explained the underlying molecular mechanisms. In 1993, Kozka et al. observed that the downregulation of GLUT4 in cultured rat adipocytes after chronic insulin treatment was alleviated by metformin [48]. In a similar study on rat adipocytes, IR induced by chronic insulin treatment was reversed by supplementing metformin in the culture medium [49]. Additionally, in metabolic syndrome-induced rats, 4-week treatment with metformin resulted in a significant decrease in IR and a significant increase in GLUT4 expression in a mixture of heart, liver, and visceral adipose tissues [50]. In a similar study protocol in diabetic mice, metformin treatment improved insulin sensitivity in vitro and increased the expression of GLUT4 in skeletal muscle [51].

Many human studies have come to similar conclusions. In support, Grisouard et al. demonstrated that in vitro 24 h incubation with metformin significantly increased glucose uptake, GLUT4 mRNA expression, and GLUT4 content in the adipocyte plasma membrane [52]. Another in vitro study observed that chronic exposure of cultured human myotubes to metformin was associated with direct enhancement of insulin action, and the increase was correlated with increased GLUT4 mRNA expression [53]. Furthermore, in a study including 35 women with PCOS who received either metformin or rosiglitazone for 6 months, GLUT4 mRNA expression in adipose tissue increased significantly in both groups, with a significant improvement in HOMA-IR [54].

On the other hand, Ciaraldi et al. conducted a study on T2DM subjects who failed glyburide therapy and were randomized to receive additional treatment with metformin or troglitazone for 3–4 months [55]. Metformin treatment increased insulin-stimulated whole-body glucose disposal rates by 20%; however, it did not affect GLUT1 and GLUT4 levels in adipocytes [55]. Another study examined the effect of 26-week metformin treatment in skeletal muscle of moderately obese subjects with newly diagnosed T2DM and found no differences in the insulin-mediated whole-body and leg muscle glucose uptake and unchanged expression of GLUT4 mRNA [56].

Interesting results were obtained in studies investigating GLUT4 expression in the endometria of PCOS patients, which is another example of tissue with IR [57]. For instance, a study by Carvajal et al. measured higher GLUT4 mRNA and protein levels in the endometria of PCOS patients taking metformin for at least 3 months than in patients not on metformin, reaching similar levels to those in the control group [58]. Additional evidence that metformin may improve endometrial IR was provided in a study where obese women with PCOS were given metformin for 3 months and were then compared to obese subjects without PCOS [59]. The endometrial GLUT4 protein and mRNA levels were lower in the PCOS group than in the control group, and the expression of both significantly increased after metformin treatment but was still lower than that of the control group [59]. A few years later, Li et al. observed downregulated levels of endometrial GLUT4 in PCOS conditions, and further in vitro experiments supported the results from previous studies that metformin induces GLUT4 expression [60]. Additional perspective can be gained from a study reporting that metformin could reverse the decreased endometrial GLUT4 expression caused by high testosterone levels (simulating hyperandrogenemia in PCOS) in cultured endometrial cells from non-PCOS women [61].

## 3. Mechanisms of the Metformin’s Effect

**Figure 1:**
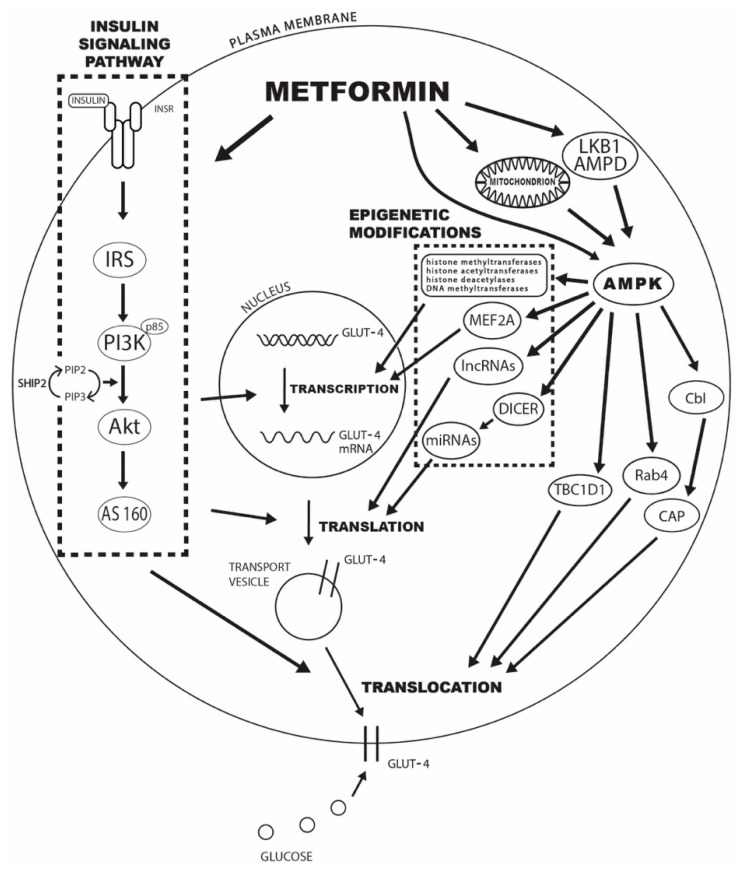
The potential mechanisms of metformin’s effect on increased GLUT4-mediated glucose transport include changes in insulin signaling pathway mediators, AMPK activation, epigenetic modifications, and enhancements in GLUT4 trafficking and translocation to the plasma membrane. **Legend:** INRS—insulin receptor; IRS—insulin receptor substrate; PI3K—phosphatidylinositol 3-kinase; Akt—protein kinase B; AS160—Akt substrate of 160 kDa; SHIP2—inositol-5-phosphatase 2 with Src homologous domain 2; PIP2—phosphatidylinositol-3,4-diphosphate; PIP3—phosphatidylinositol-3,4,5-triphosphate; AMPD—AMP deaminase; AMPK—adenosine 5′-monophosphate-activated protein kinase; MEF2A—myocyte enhancer 2A; lncRNAs—long noncoding RNAs; miRNAs—microRNAs; TBC1D1—TBC domain family, member 1; CAP—Cbl-associated protein.

The underlying mechanisms behind the observed effects of metformin treatment on GLUT4-mediated glucose transport can be divided into changes in insulin signaling pathway mediators, AMPK activation, epigenetic modifications, and enhancements in GLUT4 trafficking and translocation to the plasma membrane. The specific mechanisms are explained in detail in the following sections, and the schematic presentation can be seen in Figure 1.

### 3.1. Insulin Signaling Pathway

The insulin signaling pathway needs to be explored when analyzing the effects of metformin treatment on glucose uptake. The binding of the insulin molecule to the α subunit of the insulin receptor (INSR) causes its dimerization and the activation of the intrinsic kinase activity of the β subunits, leading to their autophosphorylation. Activated INSR acts on adaptor proteins from the insulin receptor substrate family (IRS1, IRS2, and IRS3) in a further cascade. Of particular importance are IRS1 and IRS2, which bind to the p85 regulatory subunit of phosphatidylinositol 3-kinase (PI3K) and activate it through phosphorylation. Lipid products of PI3K catalyze the formation of phosphatidylinositol-3,4,5-triphosphate (PIP3). PIP3 then binds to phosphatidylinositol-3,4,5-phosphate kinase 1 (PDK1) and protein kinase B (Akt) and attracts Akt to the cell membrane, where PDK1 phosphorylates Akt at Thr^308^. Additional phosphorylation at Ser^473/474^ is required for the complete activation of Akt. Activated Akt, through its mediator Akt substrate of 160 kDa (AS160), represents a crucial step in the regulation of the kinase cascade involved in the translocation of GLUT4 from intracellular transport vesicles to the plasma membrane [23,31,32,62]. By studying the effects of their inhibition and overexpression, a lot of evidence has been obtained suggesting that PI3K and Akt activation are essential for insulin-stimulated GLUT4 translocation [63,64]. The initial stages of the signaling pathway, especially signaling via IRS, PI3K, and Akt, have been described as crucial for the development of IR, and decreased expression of PI3K, Akt, and GLUT4 has been reported in different tissues of IR patients [32,55,65,66,67,68,69].

One of the first studies of metformin’s effect on insulin signaling mediators demonstrated that chronic insulin treatment reduced INRS tyrosine phosphorylation, PI3K activity, and Akt activity by 60–70% in cultured rat adipocytes, and these effects were prevented by the inclusion of metformin in the culture medium [49]. A later study in C2C12 cells reached similar conclusions and suggested that chronic insulin treatment drastically reduced insulin-stimulated tyrosine phosphorylation of INRS and IRS1 as well as PI3K activity, and treatment with metformin was able to reverse these changes [70]. Rice et al. demonstrated that incubation with metformin increased IRS1 mRNA and protein expression in human granulosa cell culture, as well as IRS2 protein expression [71]. In this study, as well as in a similar study by Sonntag et al., metformin incubation contributed to the additional activation of Akt in the presence of insulin via PI3K activation [71,72]. Furthermore, in ovarian tissue of rats exposed to a hyperandrogenic environment in fetal development with consequent ovulation disorders, markedly lower levels of INSR, IRS1, IRS2, and GLUT4 mRNA and proteins were found, and incubation with metformin increased mRNA and protein expression and the activation of most of these mediators [73]. Another study using the rat PCOS model demonstrated a significant reduction in IRS2 and PI3K expression, and 4-week in vivo metformin treatment significantly increased their expression [74]. Furthermore, in an additional study, reduced PI3K and Akt expression observed in ovarian and hepatic tissues of the rat PCOS model increased significantly after metformin treatment [75]. In a study by Ferreira et al., both metformin and insulin increased PI3K and GLUT4 expression in endometrial cell culture, and their combined effect was even more pronounced [67]. In contrast to insulin, metformin reduced the rate of Akt phosphorylation at Ser^473^ in this study [67].

However, some studies reported opposing results. For example, in a study by Ma et al., 24 h incubation of human granulosa cell culture with metformin resulted in reduced IRS1 mRNA and protein expression [76]. Moreover, in a study by Ciaraldi et al., unchanged levels of IRS1, GLUT1, GLUT4, p85, and Akt proteins were observed in adipocytes of patients with T2DM after 3-4 months of metformin treatment [55]. Similarly, insulin signaling parameters were unchanged after 26 weeks of metformin treatment in newly diagnosed T2DM patients [56]. Furthermore, Kim et al. studied the effect of 3-4 months of metformin treatment in T2DM subjects who failed glyburide treatment and noted no effect on basal or insulin-stimulated IRS1-associated PI3K or Akt activity in muscle cells. Additionally, protein expression of IRS1, the p85 subunit of PI3K, and Akt was unaltered after treatment [77].

Additional perspective can be gained from an in vitro study of the effects of metformin on human podocytes and a mouse skeletal muscle cell line, which demonstrated an essential role for inositol-5-phosphatase 2 with Src homologous domain 2 (SHIP2) [78]. SHIP2 hydrolyzes PIP3 back to phosphatidylinositol-3,4-diphosphate (PIP2), which is the opposite of the action of PI3K in the insulin signaling pathway. SHIP2 is overexpressed in IR tissues, and in this study, direct binding of metformin to SHIP2 and decreased activity of the phosphatase domain of SHIP2 were found [78]. At the same time, no effect of metformin was observed on the level of Akt phosphorylation [78].

### 3.2. AMPK Activation

AMPK is a serine/threonine-specific protein kinase, acting as a highly conserved master regulator of metabolism [79]. It is regulated by multiple upstream signals and affects numerous downstream substrates [80]. It exists as a trimeric complex consisting of a catalytic subunit (α-subunit) and two regulatory subunits (β- and γ-subunits) [80]. The N-terminus of the α-subunit comprises the kinase domain, and the phosphorylation of a conserved threonine (referred to as Thr^172^) in the kinase domain is required for the full activation of AMPK [80]. An increase in AMPK activity was associated with quickly increased glucose uptake and translocation of GLUT4 to the plasma membrane [52,63,81,82,83,84,85,86,87,88]. Zhou et al. were one of the first groups to propose AMPK phosphorylation and activation as a critical pathway for metformin’s pleiotropic effects [63]. The hypothesis of the metformin–AMPK–GLUT4 pathway was established; however, the exact mediators remain the subject of debate. Although metformin treatment has been coupled to AMPK activation and upregulation of glucose uptake separately, only a few studies have measured both AMPK activation and glucose uptake [63,89,90,91,92,93].

One of the most frequently invoked mechanisms of metformin action is the inhibition of mitochondrial complex I and the consequent reduction in the [ATP]:[ADP] and [ATP]:[AMP] ratios [80,82]. Due to the transient changes in cellular energy status, ATP is replaced with either ADP or AMP, allosterically activating AMPK [82,94]. Metformin has also been shown to activate AMPK through phosphorylation at Thr^172^ of the α-subunit independently of changes in adenine nucleotides [82,95,96,97]. In addition, LKB1 was identified as an upstream kinase responsible for phosphorylating and activating AMPK and was implicated as a major target of metformin [82,98,99]. However, metformin probably does not directly activate either LKB1 or AMPK since it did not influence the phosphorylation of AMPK by LKB1 in a cell-free assay [63,100]. Metformin may also interact directly with the γ-subunit of the AMPK complex, producing a structural change that promotes activation [101]. In a more novel approach, inhibition of AMP deaminase (AMPD) has been proposed as alternative mechanism to AMPK activation [102], and one report suggested that metformin may control AMP levels via effects on AMPD [103].

A study on mouse soleus muscle showed that chronic, but not acute, in vivo metformin treatment enhanced insulin-stimulated glucose uptake through activation of AMPK without notable changes in protein expression of insulin signaling mediators or GLUT4 [89]. An in vitro study in human adipocytes obtained from surgical biopsies showed that metformin incubation for 24 h increased glucose uptake, GLUT4 mRNA expression, and cellular protein level; however, suppression of metformin-induced AMPK activity by AMPKα1 silencing reduced the observed effects [52]. An approximately twofold increase in AMPK activity, independent of the AMPK level or glycemia, was also shown in adipose tissue of patients with T2DM treated with metformin for 10 weeks compared with gliclazide therapy [104]. The amount of GLUT4 protein was unaltered, and metformin also did not significantly alter the levels of insulin signaling pathway mediators [104]. These results were additionally confirmed in 3T3-L1 adipocytes [104]. However, at the time of recruitment, 12 out of 20 participants were already taking metformin, and the study had a six-week run-in period when medication was discontinued. The duration of this run-in period is important in light of a recent study that concluded that six months after metformin withdrawal, GLUT4 mRNA expression in subcutaneous adipose tissue of PCOS patients remained stable [105]. Another study in patients with T2DM showed that metformin treatment for 10 weeks significantly increased AMPK activity in skeletal muscle, and the observed effect was associated with phosphorylation of AMPK at Thr^172^ and resulted in enhanced peripheral glucose uptake [92]. Furthermore, in the endometria of PCOS patients, metformin increased GLUT4 mRNA and protein levels [58]. It significantly increased the phosphorylation and, therefore, the activity of both AMPKα and myocyte enhancer 2A (MEF2A), a transcription factor with a known binding site on the promoter region of the GLUT4 gene [58,106].

Some studies have also demonstrated the importance of metformin’s action through AMPK activation and its downstream effect on GLUT4 translocation. Activated AMPK can phosphorylate TBC1D1 (TBC domain family, member 1), which controls the translocation and plasma membrane levels of GLUT4 [80,107]. Lee et al. showed the critical role of AMPK in GLUT4 translocation in the C2C12 skeletal muscle cell line [108]. Two years later, Lee and colleagues published another study demonstrating the importance of AMPK in GLUT4 translocation in 3T3-L1 preadipocyte cells, and this effect was attenuated by AMPK knockdown [109].

### 3.3. Epigenetic Modifications

In an effort to better understand the etiology of IR conditions, the focus has now shifted from genome-wide and candidate gene association studies to the epigenetic mechanisms that could explain the dynamic and well-observed interplay between known genetic and environmental factors [110,111,112]. Therefore, a hypothesis that at least some of metformin’s effects are mediated through different epigenetic modifications was proposed, and the evidence supporting it has grown in recent years.

Treatment with metformin might influence the activity of numerous epigenetic modifying enzymes, mainly through the promotion of phosphorylation and hence activation of AMPK [113,114]. Activated AMPK can phosphorylate multiple epigenetic enzymes, such as histone acetyltransferases, class II histone deacetylases, and DNA methyltransferases [113]. For example, a study by McGee et al. reported that AMPK activation decreased the transcriptional repressor histone deacetylase 5 (HDAC5), with a known association with the GLUT4 gene, resulting in increased GLUT4 expression in human myotubes [115]. In addition, metformin has also been reported to decrease the expression of multiple histone methyltransferases and to increase the activity of the class III histone deacetylase SIRT1 [113]. The importance of such mechanisms can be seen in a study in which 3T3-L1 adipocytes with knocked down SIRT1 showed inhibited insulin-stimulated glucose uptake and impaired GLUT4 translocation [116].

Furthermore, due to the consistent findings of the importance of posttranscriptional modifications of GLUT4 mRNA and the previously established association of altered microRNA (miRNA) levels with diabetes, IR, and inflammation, the effect of metformin on various miRNAs has also been studied in recent years [117,118]. miRNAs are small noncoding regulatory RNAs that act as negative posttranscriptional regulators of gene expression [119,120]. Alterations of miRNA expression by metformin may be partially explained by an increase in DICER, one of the critical enzymes in miRNA processing [113]. Increases in DICER protein levels have been reported in metformin-treated diabetic humans and mice and also in cells treated with the direct AMPK activator 5-aminoimidazole-4-carboxamide riboside (AICAR), suggesting that AMPK activation is the primary mechanism [113,121,122,123].

Numerous miRNAs have been reported to directly or indirectly regulate insulin sensitivity and GLUT4 expression, and their levels were shown to be altered in IR conditions [101,119,124,125,126,127,128,129]. Treatment with metformin has been associated with changes in several miRNAs, with many of them having a known effect on glucose metabolism. In a model of high-fat diet rats and IR skeletal muscle L6 cells, metformin dose-dependently decreased miR-21 expression, consequently improving skeletal muscle IR [130]. A recent study examined the effect of metformin on miR-223 expression and the amount of Akt and GLUT4 proteins in IR 3T3-L1 adipocyte cells and adipocytes of diabetic patients [101]. MiR-223 was overexpressed in both, and incubation of the cell line and 3-month treatment of patients with metformin decreased its expression while increasing Akt and GLUT4 expression [101]. Mensà et al. found that miR-146a levels show a significant age-related decline that is even more notable in T2DM patients [131]. They also observed that miR-146a was significantly overexpressed in T2DM patients treated with metformin [131]. Although miR-146a is more commonly associated with inflammation [132], it has also been associated with IR and poor glycemic control in Asian Indian T2DM patients [133]. Furthermore, a randomized, double-blinded, and placebo-controlled three-month trial of metformin treatment in T2DM patients found that metformin (but not placebo) led to significant changes in circulating miR-192, miR-140-5p, and miR-222, in parallel to decreased fasting glucose and HbA1c [134]. More recently, the same concept of metformin’s effect on extracellular miRNAs was demonstrated by Ghai et al. [135]. They observed that the concentrations of several miRNAs were increased in T2DM, but they decreased to the normal range after metformin treatment. However, at present, there is little evidence that these circulating miRNAs are involved in GLUT4 expression or IR [135]. In a study in PCOS patients, 12-month metformin treatment reduced serum levels of miR-122, miR-223, and miR-29a, all of which have previously been reported to influence glucose metabolism [136].

A new area of research of metformin’s mode of action is likely to expand to other epigenetic mechanisms. In 2020, García-Calzón et al. published a paper where they discovered that by measuring blood-based epigenetic markers in the form of DNA methylation of 11 specific loci in drug-naive patients with T2DM, they could discriminate between glycemic responders and nonresponders to metformin, providing some further evidence into the role of DNA methylation [137]. Additionally, there has been limited research covering the role of long noncoding RNAs (lncRNAs) to date. In C2C12 myotubes, metformin reduced medium glucose concentration in the culture medium, increased levels of GLUT4 in the plasma membrane, and repressed lncRNA Dreh expression [138]. Interestingly, knockdown of Dreh had even more profound results, while its overexpression attenuated the glucose-lowering effect of metformin, suggesting that glucoregulatory actions of metformin are mediated in part by the lncRNA Dreh [138]. In gastric cancer cells, metformin treatment significantly inhibited the cellular functions of cancer cells, and lncRNA H19 was a crucial component in that process [139]. Interestingly, the same lncRNA H19 was previously shown to improve IR in skeletal muscle, although the proposed mechanism involves reducing ectopic lipid accumulation, not GLUT4 expression [140].

### 3.4. GLUT4 Trafficking and Translocation

Many studies have suggested that defects in GLUT4 translocation are closely related to IR [141,142,143,144,145]. The stimulation of glucose uptake requires the translocation of GLUT4 from tubulovesicular structures, named GLUT4 storage vesicles (GSV), to the cell surface, rapidly increasing the GLUT4 density at the plasma membrane and consequently glucose uptake [119]. It is well documented that insulin enhances GLUT4 translocation through incompletely defined intracellular signaling pathways involved in releasing GSVs from intracellular retention, their trafficking, tethering, and, finally, docking and fusion to the plasma membrane [7,52,119,146,147,148,149,150]. Furthermore, studies from the 1990s onwards suggested that metformin ameliorates IR independently of GLUT4 protein synthesis, mainly through subcellular redistribution of GLUT4 [49,147,151,152,153,154,155,156]. However, the mechanisms by which metformin acts on GLUT4 translocation are still largely unknown.

The understanding of glucose uptake involves the interplay between the insulin signaling pathway and GLUT4 membrane trafficking at the cellular level. Not surprisingly, identifying the molecules that link them has been a significant research focus. Since metformin has a known effect on the insulin signaling pathway, this could also explain the noted effects of metformin treatment on the GLUT4 translocation process [148]. The lipid products of PI3K contribute to the activation of Akt and atypical protein kinases C zeta and lambda (PKCζ/λ) [55,119]. The discovery in 2003 that a RabGAP, AS160, is a highly insulin-responsive Akt target provided one of the first links between insulin signaling and GLUT4 translocation, given that a significant function of Rab GTPases is to regulate vesicle traffic [1,119,148,157]. AS160 has emerged as a negative regulator in the insulin transduction relay since overexpressing a phosphorylation-defective mutant reduced insulin-dependent GLUT4 translocation, and, conversely, deletion of AS160 elevated GLUT4 levels in the plasma membrane in the absence of insulin stimulation [1,158]. Moreover, Rice et al. demonstrated that metformin increased insulin-stimulated translocation of GLUT4 to the plasma membrane in human ovarian granulosa cells via a mechanism involving PI3K activation of Akt [71]. Additionally, Polianskyte-Prause et al. demonstrated a novel mechanism of metformin action through a reduction in SHIP2 activity, since in SHIP2-overexpressing myotubes, metformin ameliorated reduced glucose uptake by slowing down GLUT4 endocytosis [78]. In addition to this, a step-by-step review of the protein related to the GLUT4 translocation machinery in skeletal muscle reported that several miRNAs have been potentially correlated with some target genes and could present additional targets of metformin action [119].

Some authors also demonstrated the crucial role of AMPK activation and its downstream effect on GLUT4 translocation mediators after metformin treatment. For example, Lee et al. noted metformin’s activity through the phosphorylation of Cbl and stimulated expression of Cbl-associated protein (CAP) in an AMPK-dependent manner, and Cbl/CAP-associated multicomplex formation modulated GLUT4 translocation in 3T3-L1 cells [109]. In another paper, Lee et al. demonstrated that metformin induced Rab4 expression via the AMPK pathway and demonstrated that the activities of AS160 and PKCζ are involved in metformin-induced Rab4 regulation in C2C12 cells [108]. Together, these studies suggest that metformin also induces Rab4 expression via AMPK–AS160–PKCζ and modulates insulin-mediated GLUT4 translocation [108]. On the other hand, Kristensen et al. did not note any changes in insulin signaling upon Akt and AS160 protein expression or their phosphorylation or changes in protein expression of Rab4 after two weeks of metformin treatment in mouse soleus muscle [89].

## 4. Conclusions

IR is a clinical condition shared by many diseases other than prediabetes and T2DM, such as obesity, PCOS, and nonalcoholic fatty liver disease. Therefore, the comprehension of the mechanisms responsible for impaired insulin action is fundamental in the attempt to ameliorate IR and to account for the favorable effects of insulin sensitizers. Although we do not know how to explain all of the mechanisms of metformin action at the molecular level, metformin, active at all sites of impaired insulin action, has been a first-line drug to improve IR for decades. The observed increase in peripheral glucose utilization after metformin treatment most likely derives from the induction of GLUT4 expression and its enhanced translocation to the plasma membrane. Based on the current data, metformin achieves this effect through direct and indirect effects on mediators from the initial stages of the insulin signaling pathway, AMPK activation, GLUT4 trafficking and translocation mediators, and complex AMPK-dependent and -independent epigenetic modifications. A better understanding of the mechanisms behind its clinically relevant increase in insulin sensitivity could help researchers and clinicians studying and treating the dramatic rise in IR conditions worldwide.

## Data Availability

Not applicable.
